# 

**Published:** 1889-07-06

**Authors:** 


					214 THE HOSPITAL. July 6, 1889.
NOTICE TO CORRESPONDENTS.
All MS., Letters, Books for Review, and other matters intended for the
Editor, should be addressed The Editor, The Lo?oe, Porchester
Square, London, \V.
Notice to Advertisers and Subscribers.
All Advertisements, Orders for Copies of The Hospital, and Business
Communications should be addressed to The Publisher, not to the Editor,
THE Hospital, 139-140, Salisbury-court, Fleet-street, E.C.
Yol. V., now ready, 4s., post free. Cases for binding, may be had of
the Publisher, at Is. 6d. each.
To Residents, Secretaries, and Matrons.
The Editor will be glad to have the Regulations and each Report as
issued by Asylums, Hospitals, Convalescent and Nursing Institutions, as
well as those of other Charities, and an early copy in duplicate of all
Appeals and Festival Notices, etc., addressed to The Editor, The Lodge,
I'trchester-square, London, W. Any superintendent, secretary, matron,
or other chief official who regularly sends such information may be
registered, on application to the Publisher, to receive free of cost a cover
for binding The Hospital in half-yearly volumes.
Amusements and Relaxation.
Second Quarterly Word Competition Commences
July 6th, ends September 28th.
Ihree Prizes of 15s., 10s., 5s., will be given for the largest number of
words derived from the words set for dissection.
Proper names, abbreviations, foreign words, words of less than four
letters, and repetitions are barred ; plurals, and past and present par-
ticiples of verbs, are allowed. Nuttall's Standard dictionary only to be
?used.
N.B.?Word dissections must be sent in WEEKLY, arranged alpha-
- betically, with correct total affixed.
The word for dissection for this, the First week of the quarter, being
"WINDSOR."
Results of Twelfth Week.
Names. June 27th.
.'Patience  108
Nurse Duty  109
Lightowlers .... 112
Scrooge   ?
Reldas  ?
Tinie   10/
K. G  79
Broxbourne  107
Lauriston  110
Spes  98
Gretta  ?
1'rancesca  ?
Heatherbell  ?
Sycamore
Arctus   _
Amicus   105
Scratcher
Jemima
? Concours....
c. j.s ; _
See-Saw   93
Itosemont  -
Jenny Wren ...! 93
Eric
Names. June 27th
Tiny  82
N'importe Qui .. 67
Polite   51
W. G. E  -
Robe   ?
Beryl   ?
Daffodil   ?
A Schoolgirl.... 53
Alice R  ?
Belfast   ?
Gentian   ?
Hope   ?
Louie   ?
Hamilton   ?
E. 11  ?
F. J. D  ?
Ladyr   ?
Surgical Nurse.. ?
L. Pearce   ?
Contentio  ?
H. W  ?
Esperance  ?
H. B  ?
The names of the winners of the First Quarterly Word Competition
? will be published next week.
Notice to all Correspondents.
N.B.?All letters referring to this page which do not arrive at 139 and
140, Salisbury-court, London, E.C., by the first post on Thursdays, and
are not addressed PRIZE EDITOR, will in future be disqualified and
disregarded
Competitors can enter for all quarterly competitions, but ho com-
petitor may take more than two quarterly prizes during the year.
Conditions.
I. Every paper sent in must be clearly written, and one side only must
be used. All competitors must mark at the head of their papers the
number of their competition.
II. Each paper must be signed by the author with his or her real name
and address.. A nom de plume may be added if the writer does not desire
to be referred to by us by his real name. In the case of all prize-winners,
however, the real name and address will be published.
III. All communications relating to prize competitions should be ad-
dressed to "The Prize Editor, The Hospital, 139 and 140, Salisbury,
court, London, E.C." Competitors are requested not to include in letters,
etc., to the Prize Editor communications on matters of other business.
All letters must be fully prepaid.
IV. The Prize Editor of The Hospital is the sole judge of the com-
petitions, and his deeision is in all cases final and without appeal.
Y. The Prize Editor reserves the right to publish any paper sent in,
whether it receives a prize or not. He does not hold himself responsibly
?for any MSS. sent, nor can he undertake to return rejected competitions.
Scraps and Gleanings.
Pasteur is a member of the Royal Society.
Mrs. Black's ball in aid of her Cottage Hospital brought in ?405.
The Royal Commission on Vaccination hold their meetings in private.
A Sunday Concert is to be given at Pontefract in aid of the dis-
pensary.
The Queen has conferred the title of Royal on the Glasgow Childreu s
Hospital.
There was a garden party at the Cancer Hospital, Brompton, on
Wednesday last.
Sonenschein will shortly publish a Manual of Home Nursing by Miss
Louisa E. Dobree.
The Nineteenth Century contains an article on the last illness of Lord
Beaconsfield by Dr. Kidd.
Miss Katherine Maguire took both the medical and surgical prizes
at Adelaide Hospital, Dublin.
All four medical officers of Dewsbury Infirmary have been re-elected
for another term of ten years.
Mr. Charles Henry Taylor has been appointed house surgeon of
Derbyshire General Infirmary.
Ilfracombe kept Hospital Sunday last month. Forty pounds was
collected at the parish church.
On Hospital Sunday, at Harrow, ?51 was collected in the Parish
Church for the Cottage Hospital.
The Hospital for Incurable Children at Cheyne Walk is to be shortly
removed to new premises near Chelsea Church.
Colonel Hill, M.P., has been appointed president, and Dr. Franklen
G. Evans vice-president of the Cardiff Infirmary.
The first annual meeting of the Invalid Children's Aid Association was
held on Thursday, the Earl of Aberdeen in the chair.
The Bootle Timts complains that the house surgeon of the Borough
Hospital spends too much of his time at the police court.
Dr. Creigiiton has an article on Vaccination, and Miss Twining has .
an article on Poor Law Infirmaries, in the National Review.
The Duchess of Westminster presented certificates to the successful
students of the National Health Society's lectures last Saturday.
A patient at the Dublin Rotunda lately leaped from the window and
killed herself. It is alleged that the nurse in charge was asleep.
The site for the new infirmary at Halifax will be in Heath School-
lane. Over ?43,000 has been subscribed towards the building fund.
The amount collected last Sunday in aid of the Metropolitan Hos-
pital Sunday Fund at Emmanuel Church, Gunnersbury, was ?2119s. 3d.
There were many references to Father Damien on Hospital Sunday I
amongst others Dr. Vaughan referred to the heroism of the leper priest.
On Monday and Tuesday tableaux vivants were given at Chelsea in aid
of the local branch of the National and Metropolitan Nursing Association-
Surgeon H. F. Babington, M.B., has resigned his commission, which
bore date July 31st, 1880. He was engaged in the war against the Boers
in 1881.
Amongst the flood of letters on leprosy which lately appeared in th?
daily papers, a letter from Jonathan Hutchinson in the Evening News
was most worth reading.
Radcliffe Infirmary Governors plead that the surplus of the
Oxford Savings Bank could be better used by them, than applied to
paying off the National Debt.
Lord Randolph Churchill will preside to-night at a dinner in aid of
the funds of the Hospital for Diseases of the Throat, Golden-square,
be held at the Hotel Metropole.
Mr. Hugh Alexander, chairman of the Association of Public
Sanitary Inspectors of Great Britain, presided at the fourth provincial
meeting, held recently, at Chelmsford.
Professor Walley, of Edinburgh Veterinary College, has been
presented with a gold medal in recognition of his services in connection
with the prohibition of the dishorning of cattle.
THE Newcastle Hospital Sunday Fund amounts to ?4,435 lis. 7d.,.?*
which sum ?1,924 3s. 3d. was collected at manufactories, showing an in'
crease from this source of ?258 6s. 3d. over that of the previous year.
The Society of Friends lias decided to admit to its chief boarding-
school any children of its members, even if the children should be un'
vaccinated. This is the result of some agitation over a period of years.
The British Medical Journal states that a Bill has been drafted W
the Legislative Council of India on the leprosy question?including
segregation and medical care?and will shortly be brought forward f?r
discussion.
The tenth annual house-to-house collection in aid of the funds of th?
Lincoln County Hospital was made in Lincoln on June 15th last. The
city was divided as usual into 49 districts, and altogether about 1?L
collectors were engaged in the praiseworthy work.
A candidate at a recent medical examination said that (1) a flower
stalk was its pedestal; (2) a premocarp consisted of two pericarps; anl
that HI was idiotic acid. He told his tutor he had answered every ques-
tion, and he could not understand why he did not pass.
Two new hospitals have just been opened in Moscow. One of these is
a lying-in clinic attached to the University, the funds for buildinfe
being given by Mdme. Pashloff. The other is an asylum for insan
patients. This, too, has been given by a lady, Mdme. Alexieff.
The St. James's Gazette says that a large hospital is badly needed i"
Leavenworth, Kansas. Last month 22,000 men of that town formauy
registered themselves as sick. In Leavenworth you have to sign a ceri
ficate declaring that you are ill before you can get a drink of liquor.
A death from hydrophobia has occurred at Great Bedwyn, neai
Devizes. A little boy named Francis Wallis, three and a half year.? p(j
age, was bitten by a dog last February, and on Sunday he was setf
with convulsions. The medical evidence at the inquest showed tn
hydrophobia was the cause of death.

				

